# Tumor-area mitosis proportion as an image-derived mitotic phenotype in lung squamous cell carcinoma: Patient-level prognostic and multi-omics evaluation

**DOI:** 10.1016/j.jpi.2026.100750

**Published:** 2026-10-06

**Authors:** Gulcin Yilmaz Gunes, Mehmet Akif Cifci

**Affiliations:** a Department of Pulmonology, Bandırma Training and Research Hospital, Bandırma, Türkiye; b Institute of Research and Development, Duy Tan University, Da Nang, Viet Nam; c School of Engineering and Technology, Duy Tan University, Da Nang, Viet Nam; d Department of Computer Engineering, Bandırma Onyedi Eylül University, Bandırma, Balıkesir 10200, Türkiye; e Engineering and Informatics Department, Klaipėdos Valstybinė Kolegija/Higher Education Institution, 92294 Klaipėda, Lithuania

**Keywords:** Lung squamous cell carcinoma, Mitosis, Transfer learning, Computational pathology, Tumor-area mitosis proportion, Deep learning

## Abstract

**Background:**

Mitotic activity is a recognized marker of tumor proliferation, yet its reproducible assessment across whole-slide images (WSIs) remains difficult. We developed a transfer-learning framework for lung squamous cell carcinoma (LUSC) and evaluated whether a patch-derived tumor-area mitosis proportion (TAMP) was associated with survival and molecular tumor characteristics.

**Methods:**

The analysis combined MIDOG 2022 with TCGA-LUSC, including 478 patients and 512 diagnostic WSIs after quality control. Tumor regions and mitotic figures were reviewed by two pathologists with interobserver agreement of Cohen's κ ≥ 0.85. A ResNet-50 model was trained on MIDOG and subsequently adapted to TCGA-LUSC using Focal Loss and stain-aware augmentation. Target-domain performance was evaluated using patient-level stratified 5-fold cross-validation and an additional WSI-level holdout analysis to address potential leakage from the initial patch-level partitioning. TAMP was calculated from patient-level out-of-fold predictions as the proportion of evaluable tumor patches classified as mitosis-positive. Associations with overall survival, immune and genomic features, transcriptomic programs, predicted drug response, and transcription-factor activity were subsequently examined. Multivariable Cox regression adjusted for age, sex, smoking status, tumor stage, and Eastern Cooperative Oncology Group performance status.

**Results:**

The model achieved an area under the receiver operating characteristic curve (AUC) of 0.975 (95% confidence interval (CI): 0.965–0.980) on the held-out MIDOG test set. In TCGA-LUSC, fine-tuning increased the initial patch-level AUC from 0.82 to 0.92. Patient-level cross-validation yielded a mean AUC of 0.89 (95% CI: 0.86–0.90), and WSI-level holdout validation yielded an AUC of 0.90 (95% CI: 0.84–0.90). High TAMP was associated with poorer overall survival (log-rank *p* = 0.008; HR = 1.82, 95% CI: 1.18–2.81) and remained associated with mortality after multivariable adjustment (adjusted HR = 1.68, 95% CI: 1.08–2.62; *p* = 0.022). Higher TAMP was also associated with lower stromal fraction, selected immune-cell differences, greater indel-derived neoantigen load and indel burden, and enrichment of stem-cell proliferation, growth-factor-response, E2F-target, G2M-checkpoint, and MYC-target programs. Additional associations were observed with predicted sensitivity to selected cell-cycle-targeting compounds and with proliferation-related transcription-factor activity.

**Conclusions:**

TAMP provides a patient-level, image-derived representation of mitosis-positive tumor regions that was associated with survival and several molecular features in TCGA-LUSC. Its association with overall survival persisted after adjustment for major clinical covariates. External multicenter validation, prespecified threshold evaluation, and prospective assessment are required before clinical use.

## Introduction

Lung cancer remains one of the leading causes of cancer-related mortality worldwide and continues to impose a substantial clinical burden.^1^, ^2^ Lung squamous cell carcinoma (LUSC), a major histological subtype of non-small cell lung cancer, accounts for approximately 25%–30% of lung cancer cases and typically originates from the bronchial epithelium of the central airways.^3^, ^4^ LUSC is strongly associated with tobacco exposure and is characterized by recurrent genomic alterations involving *TP53*, *CDKN2A*, *PIK3CA*, and other genes governing cell-cycle regulation and tumor growth.^5^ Unlike lung adenocarcinoma, however, LUSC has relatively few routinely actionable molecular alterations, limiting opportunities for genotype-directed treatment, and reinforcing the need for additional markers that can better characterize tumor aggressiveness and support prognostic assessment.^6^

Sustained cellular proliferation is a fundamental feature of malignant growth.^7^ In routine histopathology, mitotic figures provide direct morphological evidence of active cell division and have long been used as indicators of proliferative activity and biological aggressiveness.^8^ Mitotic assessment contributes to established grading or classification systems in several malignancies, including breast cancer and pulmonary neuroendocrine neoplasms.^9^, ^10^ In LUSC, increased mitotic activity has also been associated with aggressive pathological features and disruption of cell-cycle control, although it has not been incorporated into a standardized quantitative framework for routine prognostic evaluation.^11^

Conventional mitotic counting relies on manual microscopic examination and therefore remains labor-intensive and susceptible to interobserver variation.^12^ Accurate recognition may also be difficult because apoptotic bodies, hyperchromatic nuclei, pyknotic structures, and tissue-processing artifacts can resemble true mitotic figures.^13^, ^14^ These limitations become particularly important in whole-slide imaging (WSI), where exhaustive assessment of large and heterogeneous tumor regions is impractical. Conventional mitotic counts also represent observations from selected microscopic fields rather than a summary of the full evaluable tumor compartment. A computational approach that systematically evaluates mitosis-related morphology across broader tumor regions may therefore provide a more reproducible and spatially comprehensive representation of proliferative activity.

The tumor-area mitosis proportion (TAMP) examined in this study was designed to capture a complementary aspect of tumor proliferation relative to conventional mitotic counting and immunohistochemical proliferation markers such as Ki-67. Conventional mitotic counts are typically based on selected microscopic fields and may therefore be influenced by field selection and spatial heterogeneity, whereas Ki-67 reflects the proportion of cells engaged in the cell cycle rather than mitosis specifically. In contrast, TAMP summarizes the proportion of evaluable tumor image patches classified as mitosis-positive across the pathologist-defined tumor compartment. This whole-slide-derived approach has the potential to provide broader spatial sampling of mitosis-associated morphology and to reduce dependence on manually selected high-power fields. TAMP should nevertheless be interpreted as a patch-derived imaging phenotype rather than as a replacement for conventional mitotic counts or Ki-67. Its potential value lies in providing a complementary, spatially distributed representation of mitosis-associated morphology that can be derived systematically from digital WSIs. Recent advances in computational pathology and deep learning have made large-scale analysis of histopathological WSIs increasingly feasible.^15^ Convolutional neural networks have shown strong performance in automated mitotic figure recognition, whereas benchmark initiatives such as the mitosis domain generalization (MIDOG) challenges have emphasized the importance of maintaining performance across different scanners, labs, staining conditions, and tissue domains.^16^ Despite these advances, domain shift remains a major obstacle when models developed in one tissue context are transferred to another. Transfer learning provides a practical means of addressing this problem by retaining lower-level morphological representations that may remain informative across domains while adapting higher-level features to the target tissue.^17^

Computational pathology also provides an opportunity to move beyond isolated image classification and examine whether image-derived phenotypes reflect clinically and biologically relevant tumor characteristics. TCGA-LUSC combines diagnostic WSIs with matched clinical, genomic, and transcriptomic information, allowing morphological findings to be studied alongside multiple dimensions of tumor biology.^5^ The relationship between computationally derived mitotic phenotypes and overall survival, tumor microenvironment composition, genomic alterations, transcriptional programs, predicted drug response, and regulatory activity in LUSC, however, remains incompletely characterized.

In this study, we developed a transfer-learning framework using MIDOG as the source domain and TCGA-LUSC as the target domain for patch-level mitosis classification. We subsequently derived TAMP from patient-level out-of-fold predictions as the proportion of evaluable tumor-region patches classified as mitosis-positive. This design allowed the imaging phenotype to be evaluated while minimizing the influence of patient overlap between model training and downstream analysis. TAMP was then examined in relation to overall survival, tumor microenvironment characteristics, genomic features, transcriptomic pathway enrichment, predicted drug sensitivity, and inferred transcription-factor activity.

The study addressed four related objectives. First, we adapted a transfer-learning-based mitosis classifier from MIDOG to LUSC WSI patches. Second, we derived TAMP as a patient-level measure of the prevalence of mitosis-positive regions within pathologist-defined tumor tissue. Third, we evaluated whether TAMP was associated with overall survival and whether this relationship persisted after adjustment for available clinical prognostic factors, while also examining its associations with selected immune and genomic characteristics. Fourth, we integrated TAMP with transcriptomic pathway enrichment, predicted drug-response analysis, and inferred transcription-factor activity to characterize the molecular context of tumors with elevated mitosis-associated morphology. This framework extends automated mitosis analysis from local image classification toward patient-level clinicopathological and multi-omics characterization.

## Literature review

Mitotic activity is a well-established histopathological marker of tumor proliferation and aggressiveness. In several malignancies, mitotic count is incorporated directly into diagnostic or grading systems because it provides a morphological measure of active cell division. In breast cancer, mitotic activity contributes to the Nottingham histological grade.^18^ In pulmonary neuroendocrine neoplasms, mitotic count is a key criterion for distinguishing typical carcinoid, atypical carcinoid, and high-grade neuroendocrine carcinomas. Increased mitotic activity has also been associated with aggressive histological features, higher proliferative capacity, and adverse clinical behavior in non-small cell lung cancer, including LUSC.^19^ However, a standardized and reproducible framework for quantitative mitotic assessment in LUSC has not yet been established.^20^

Manual mitotic counting remains challenging in routine histopathology. The process is labor-intensive, depends on the selection of representative high-power fields, and is affected by interobserver variability.^16^ Reliable identification is further complicated by morphological mimics such as apoptotic bodies, hyperchromatic nuclei, pyknotic cells, and tissue-processing artifacts.^21^ These limitations have motivated the development of automated image-analysis methods capable of detecting mitotic figures consistently across hematoxylin-and-eosin (H&E)-stained WSIs.^22^

Early computational approaches relied primarily on handcrafted morphological, texture, and color features combined with conventional machine-learning classifiers.^23^ The emergence of deep convolutional neural networks substantially improved automated mitosis recognition by enabling direct learning of discriminative morphological representations from image data.^14^ Architectures based on residual networks, DenseNet, EfficientNet, and related convolutional backbones have demonstrated strong performance for patch-level mitosis classification.^24^, ^25^, ^26^ Nevertheless, models trained under controlled conditions frequently experience performance degradation when applied to images acquired using different scanners, staining protocols, labs, or tissue types.^27^ This domain-shift problem remains one of the major barriers to robust computational pathology systems.^28^

To improve detection accuracy and reduce false-positive predictions, subsequent studies increasingly adopted multistage architectures. These approaches commonly separate candidate generation from final mitosis classification, allowing a sensitive first stage to identify potential mitotic regions followed by a more discriminative refinement stage.^29^ Detection frameworks based on Mask R-CNN and related region-based models have shown promising performance in histological mitosis detection.^30^ RetinaNet-based approaches have also been investigated for direct detection of mitotic figures in whole-slide imagery.^31^ More recent systems have combined rapid candidate segmentation with high-capacity classification networks, improving both computational efficiency and classification performance.^32^

The MIDOG challenge series has become an important benchmark for evaluating the robustness of automated mitosis detection under domain shift.^16^ MIDOG datasets intentionally incorporate variability related to scanners, institutions, staining conditions, and tissue appearance, providing a more realistic assessment of generalizability than conventional single-center datasets.^16^ Methods developed for these challenges have explored stain augmentation and normalization strategies,^28^ adversarial domain adaptation,^31^ style-transfer approaches,^33^ Fourier-based augmentation,^34^ and self-supervised representation learning.^35^ Ensemble-based strategies have also been used to improve robustness across heterogeneous image domains.^36^

More recent work has expanded automated mitosis analysis beyond single-tumor datasets. Pan-cancer approaches have investigated whether mitotic morphology can be recognized consistently across multiple tissue types.^37^ Other studies have focused on distinguishing typical from atypical mitotic figures, reflecting increasing interest in the biological information contained in mitotic morphology itself.^38^ These developments suggest that automated mitosis analysis is evolving from a narrow detection problem toward a broader computational characterization of proliferative phenotypes.

Despite this progress, much of the existing literature remains concentrated on breast cancer or mixed-organ benchmark datasets.^13^ Dedicated automated mitosis studies in LUSC remain comparatively limited.^37^ This is important because LUSC has distinct architectural, cytological, stromal, and inflammatory characteristics that may influence the transferability of models developed in other tumor types.^20^ Large public resources such as TCGA-LUSC provide diagnostic WSIs together with extensive clinical, genomic, and transcriptomic information, creating an opportunity to study mitotic activity within a broader biological context.^5^ However, TCGA-LUSC does not provide exhaustive mitotic annotations, making direct supervised training difficult.^39^

Transfer learning offers a practical strategy for addressing this limitation. Features learned from well-annotated source-domain datasets can be adapted to target tumor types with fewer annotations, potentially reducing the annotation burden while preserving useful morphological representations.^40^ In computational pathology, lower-level features such as nuclear contours, chromatin texture, and local cellular organization may remain partially transferable across tissue types and higher-level representations can then be fine-tuned to account for the specific morphology and staining characteristics of LUSC.^41^ Cross-cancer transfer from a well-annotated mitosis dataset such as MIDOG to TCGA-LUSC is therefore a biologically and computationally motivated approach.^42^

An additional direction in computational pathology is the integration of image-derived phenotypes with clinical and molecular information.^43^ Rather than treating automated mitosis detection solely as an image-classification task, quantitative measures of mitotic activity can be investigated in relation to patient survival, genomic instability, immune composition, signaling pathways, and therapeutic vulnerability.^44^ Increased proliferative activity has been linked to cell-cycle dysregulation and genomic instability across multiple malignancies.^7^ It may also be associated with changes in immune-cell composition and tumor–microenvironment interactions.^44^ Transcriptomic analyses can further identify biological pathways enriched in highly proliferative tumors.^45^

Gene set enrichment analysis provides a systematic framework for identifying coordinated pathway-level transcriptional changes associated with quantitative tumor phenotypes.^45^ Computational drug-response approaches can additionally generate hypotheses regarding potential therapeutic vulnerabilities from tumor transcriptomic profiles.^46^ Similarly, transcription-factor activity inference methods enable investigation of regulatory programs that may underlie highly proliferative tumor states.^47^ Integrating these analyses with computationally quantified mitotic activity may therefore provide a more comprehensive view of LUSC biology.

Taken together, the literature indicates that deep-learning-based mitosis detection has achieved substantial technical progress, particularly through domain-generalization and transfer-learning strategies.^16^ However, systematic application to LUSC and integration with clinical and multi-omics characteristics remain limited.^20^ The present study addresses this gap by transferring a mitosis-classification model from MIDOG 2022 to TCGA-LUSC and deriving TAMP as a patch-derived imaging measure of the prevalence of mitosis-positive tumor regions.^48^ TAMP is subsequently evaluated in relation to overall survival, tumor–microenvironment characteristics, genomic features, pathway enrichment, predicted drug sensitivity, and inferred transcription-factor activity, extending automated mitosis analysis from image classification toward an integrated computational pathology framework.^12^

## Materials and methods

### Data acquisition and cohort selection

Two complementary digital pathology cohorts were used to develop the transfer-learning framework for mitotic figure classification and to examine the clinical and molecular correlates of image-derived mitotic activity in LUSC. MIDOG 2022 served as the source domain for model development, whereas TCGA-LUSC provided the target domain for model adaptation and subsequent clinicomolecular analyses.

The source-domain subset used in this study consisted exclusively of 150 H&E-stained WSIs of invasive breast carcinoma from MIDOG 2022. No non-breast tissue domains from the broader MIDOG resources were included in source-domain model development. The slides were acquired at 40× magnification with a nominal spatial resolution of 0.25 μm/pixel, and expert-annotated mitotic figures provided the reference labels for training. This subset was selected because it combines standardized expert annotation with substantial scanner- and stain-related variability, providing an appropriate source domain for cross-tissue adaptation to LUSC.

### Histopathological annotation and patch generation

TCGA-LUSC WSIs were independently reviewed in ASAP software (version 1.9) by two board-certified pathologists with expertise in thoracic pathology. Tumor regions of interest were manually delineated, after which unequivocal mitotic figures were annotated within the tumor compartment. Mitotic figures were identified using established morphological criteria, including condensed basophilic chromosomes, loss of an intact nuclear membrane, and morphological features consistent with active mitosis rather than apoptosis, necrosis, or other mitotic mimics.

Interobserver agreement was evaluated specifically for mitotic figure identification using Cohen's κ. Agreement exceeded the prespecified acceptance threshold of κ≥0.85. Discrepant annotations were jointly reviewed and resolved by consensus, and the resulting consensus annotations were used as the reference standard for subsequent patch generation.

The final annotation set comprised 8742 mitotic figures within 3186 tumor regions across 512 diagnostic WSIs. The number of annotated mitotic figures per slide had a median of 14 (interquartile range [IQR], 8–23; range, 2–67), whereas the number of annotated tumor regions per slide had a median of 6 (IQR, 4–9; range, 1–18).

Binary tumor masks were generated from the manually delineated regions. Non-overlapping RGB patches of 128 × 128 pixels were then extracted from the tumor masks at the native 40× resolution. At a nominal spatial resolution of 0.25 μm/pixel, each patch represented an approximately 32 × 32 μm tissue field. This patch size was selected to preserve local nuclear morphology while maintaining computational efficiency. A patch was labeled positive when the centroid of at least one consensus-annotated mitotic figure was located within its boundaries. All remaining patches within the tumor mask were labeled negative. Background and tissue-poor patches were excluded using Otsu thresholding, which determines an intensity threshold by maximizing between-class variance.^49^ This preprocessing step removed visually uninformative regions and reduced unnecessary computational processing before model training.

### Modified ResNet-50 architecture

Mitotic recognition was formulated as a binary patch-classification task using ResNet-50 as the backbone architecture. The residual design of ResNet-50 facilitates the optimization of deep convolutional representations through identity-based skip connections.^24^ For an input patch x, the network produced a scalar logit $z\left(x\right)$, which was converted to the probability of a mitosis-positive prediction using the sigmoid function:

$$P\left(y=1|x\right)=\sigma \left(z\right)=\frac{1}{1+e^{-z}}.$$

The network began with a 7×7 convolutional layer containing 64 filters with stride 2, followed by 3×3 max pooling. The feature-extraction backbone comprised four residual stages containing 3, 4, 6, and 3 bottleneck blocks, respectively. Each bottleneck block consisted of a 1×1 convolution for channel reduction, a 3×3 convolution for spatial feature extraction, and a 1×1 convolution for channel expansion. Batch normalization and rectified linear unit activation were applied within the residual blocks, whereas skip connections preserved information across successive transformations. The four residual stages produced feature representations with 256, 512, 1024, and 2048 output channels, respectively. Global average pooling reduced the final convolutional representation to a 2048-dimensional feature vector, which was passed to a single-neuron fully connected classification layer.

ResNet-50 was selected to provide greater representational depth than shallower residual architectures while maintaining a computationally tractable convolutional framework. Its bottleneck structure enables hierarchical extraction of increasingly complex morphological features and was therefore considered appropriate for learning transferable representations of mitotic morphology across the MIDOG source domain and TCGA-LUSC target domain.

### Two-stage transfer learning

A two-stage transfer-learning strategy was used to address morphological and staining differences between the MIDOG breast-carcinoma source domain and the TCGA-LUSC target domain. In the first stage, the complete ResNet-50 network was trained on MIDOG patches to learn transferable representations of mitotic morphology, including chromatin condensation, nuclear contour, texture, and local cellular context. In the second stage, the network was adapted to TCGA-LUSC. The initial convolutional layer, max-pooling layer, Stage 1, and Stage 2 were frozen, whereas Stages 3 and 4 and the final classification layer remained trainable. This configuration preserved lower-level morphological representations learned from the source domain while allowing higher-level features to adapt to LUSC-specific cellular and tissue characteristics.

For the initial assessment of target-domain adaptation, TCGA-LUSC patches were stratified into 70% training, 15% validation, and 15% test partitions while preserving class proportions. This initial patch-level partition was used to quantify the effect of LUSC-specific fine-tuning. Because patches originating from the same patient could occur in different partitions, patient overlap was subsequently assessed. To obtain a leakage-mitigated estimate of target-domain performance, stratified 5-fold cross-validation was therefore implemented at the patient level, as described in the Data leakage assessment and mitigation section. Within each fold, all patches originating from a given patient were assigned exclusively to either the training or held-out evaluation set, preventing patient-level overlap between model development and evaluation. An additional WSI-level holdout analysis was performed as a complementary robustness assessment.

Optimization was performed for a maximum of 30 epochs using the Adam optimizer with a learning rate of $1\times 10^{-4}$, weight decay of $1\times 10^{-5}$, and a batch size of 32. Early stopping was applied when the validation loss failed to improve for five consecutive epochs. The ResNet-50 architecture, optimization parameters, augmentation procedure, and fine-tuning strategy were held constant across the patient-level cross-validation folds to ensure directly comparable performance estimates.

### Class-imbalance handling and data augmentation

Mitotic patches represented approximately 3% of the available TCGA-LUSC patch population, creating substantial class imbalance. We therefore used Focal Loss, which reduces the contribution of easily classified examples and increases the relative influence of difficult minority-class observations.^50^ For binary classification, the loss was expressed as:

$$\;L_{\mathrm{FL}}=-\alpha_{t}{\left(1-p_{t}\right)}^{\gamma }\log\left(p_{t}\right),\;\;$$

where $p_{t}$ denotes the predicted probability assigned to the correct class, $\alpha_{t}$ controls class weighting, and γ modulates the down-weighting of well-classified examples. We used α=0.25 and γ=2.0.

Training-only augmentation was implemented with Albumentations.^51^ Geometric transformations included horizontal and vertical flips, 90° rotations, and affine transformations with rotation within ±15°, translation up to 10% of patch dimensions, and scaling between 0.90 and 1.10. Color variability was introduced in HSV space using hue shifts of ±18 and saturation/value shifts of ±20. Gaussian noise with variance sampled from 5.0 to 30.0 and Gaussian blur with kernel sizes of 3 × 3 or 5 × 5 and σbetween 0.1 and 2.0 were applied with probability 0.3.

All images were normalized using ImageNet channel statistics. Validation and test patches underwent normalization only and were not augmented.

### Performance evaluation and model interpretation

Model performance was evaluated using the area under the receiver operating characteristic curve (AUC), sensitivity, specificity, precision, accuracy, recall, and F1-score. For a classification threshold t, the F1-score was calculated as:

$$F1=2\frac{\text{Precision}\times \text{Recall}}{\text{Precision}+\text{Recall}}.$$

The final operating threshold was determined from the validation set by maximizing the Youden index,

$$J\left(t\right)=\text{Sensitivity}\left(t\right)+\text{Specificity}\left(t\right)-1.$$

Source-domain performance was evaluated on the held-out MIDOG test set. Following target-domain fine-tuning, performance was initially assessed on the TCGA-LUSC patch-level validation and test partitions. Because patch-level partitioning can place patches from the same patient in different subsets, additional patient- and slide-level validation procedures were performed as described in the Data leakage assessment and mitigation section.

Model interpretability was examined using Gradient-weighted Class Activation Mapping (Grad-CAM).^52^ Gradients associated with the mitosis-positive output were propagated to the final convolutional layer to identify image regions contributing most strongly to each prediction.

#### Data leakage assessment and mitigation

Potential information leakage arising from the initial patch-level partitioning of TCGA-LUSC was assessed by tracing each extracted patch to its originating patient. Among 478 patients, 142 (29.7%) contributed patches to more than one data partition, including 67 patients (14.0%) represented in both the training and test sets. This overlap indicated that the original patch-level evaluation could provide an optimistically biased estimate of generalization performance.

To obtain a patient-independent performance estimate, we therefore implemented stratified 5-fold cross-validation at the patient level. All patches originating from a given patient were assigned exclusively to 1-fold, preventing the same patient from contributing data to both model training and held-out evaluation within any cross-validation iteration. The model was re-trained separately for each fold using the architecture, optimization parameters, augmentation procedures, and fine-tuning strategy specified in the Modified ResNet-50 architecture, Two-stage transfer learning, and Class-imbalance handling and data augmentation sections.

Patient-level cross-validation yielded a mean AUC of 0.89 (95% confidence interval (CI): 0.86–0.90), compared with an AUC of 0.92 under the original patch-level partitioning. The absolute decrease of 0.03, corresponding to approximately 3.3% relative reduction, indicates modest performance inflation associated with patient overlap. Nevertheless, the leakage-mitigated AUC remained higher than the AUC of 0.82 obtained when the MIDOG-trained model was applied to TCGA-LUSC before target-domain fine-tuning, supporting a genuine improvement following LUSC-specific adaptation.

As an additional robustness assessment, a separate WSI-level holdout analysis was performed in which 20% of WSIs were excluded from model training and reserved for evaluation. Because some TCGA-LUSC patients contributed more than one diagnostic WSI, this slide-level analysis was considered complementary to, rather than independent of, the patient-level cross-validation. The patient-level 5-fold cross-validation therefore served as the primary leakage-controlled estimate of target-domain performance. The WSI-level holdout yielded an AUC of 0.90 (95% CI: 0.84–0.90).

### Tumor-area mitosis proportion

The fine-tuned network was used to derive a quantitative imaging measure of mitosis-positive tumor regions across TCGA-LUSC. For all downstream analyses, predictions were generated within the patient-level 5-fold cross-validation framework described in the Data leakage assessment and mitigation section. Each patient therefore received out-of-fold predictions from a model that had not been trained on patches from that patient. Tissue-containing tumor patches were assigned a mitosis probability, and patches exceeding the validation-derived Youden threshold were classified as mitosis-positive.

For slide s, TAMP was defined as $\mathrm{TAM}P_{s}=\frac{N_{s}^{+}}{N_{s}^{\text{tumor}}}\times 100,$ where $N_{s}^{+}$ denotes the number of tumor-region patches classified as mitosis-positive and $N_{s}^{\text{tumor}}\;$denotes the total number of evaluable patches within the pathologist-defined tumor mask. TAMP was retained as the primary imaging phenotype because restricting the denominator to the tumor compartment reduces dilution from adjacent normal and other non-neoplastic tissue. Importantly, TAMP represents the proportion of tumor patches classified as mitosis-positive by the model and is not equivalent to a conventional manually enumerated mitotic count per high-power field. It should therefore be interpreted as a patch-derived measure of the extent of mitosis-associated morphology within the evaluable tumor compartment rather than as a direct count of individual mitotic figures.

For patients with more than one diagnostic WSI, slide-level measurements were aggregated to obtain a patient-level TAMP value. Patients were then categorized into high- and low-TAMP groups using the cohort median for survival and molecular comparisons. All survival, genomic, transcriptomic, immune, regulatory, and predicted drug-response analyses were performed using these patient-level out-of-fold TAMP estimates rather than predictions generated from the original patch-level partitioning.

### Transcriptomic and pathway analysis

RNA-sequencing data matched to the TCGA-LUSC imaging cohort were analyzed to characterize transcriptional programs associated with patient-level TAMP. Differential expression between high- and low-TAMP tumors was assessed using DESeq2,^53^ which models RNA-sequencing count data through negative-binomial generalized linear models.

Gene Set Enrichment Analysis was subsequently performed with clusterProfiler using Gene Ontology, KEGG, and Hallmark gene-set collections from MSigDB. Genes were ranked according to the differential-expression statistic, and enrichment was evaluated across the complete ordered gene list rather than by applying an arbitrary significance threshold to individual genes. Pathways with a Benjamini–Hochberg false-discovery rate below 0.05 were considered statistically significant.

This analysis was designed to determine whether differences in the image-derived TAMP phenotype were accompanied by coordinated transcriptional programs. Particular attention was given to pathways related to cellular proliferation, cell-cycle regulation, stem-cell processes, growth-factor response, and mitotic control. Enrichment results were interpreted as associations between TAMP and transcriptional states and not as evidence of direct pathway activation or causal regulation. All transcriptomic analyses were based on the patient-level out-of-fold TAMP estimates defined in the Tumor-area mitosis proportion section.

### Predicted drug response

Transcriptome-based drug-response estimates were generated using oncoPredict.^46^ This approach transfers drug-response relationships learned from pharmacogenomic reference datasets to patient tumor-expression profiles and produces predicted half-maximal inhibitory concentration values for individual compounds.

For drug d and patient i, the predicted response was represented as:

$${\hat{\mathrm{IC}}}_{50}^{\left(i,d\right)}=f_{d}\left(x_{i}\right),$$

where $x_{i}$ denotes the transcriptomic profile of patient i, and $f_{d}$ represents the drug-specific predictive function derived from the pharmacogenomic reference data.

Predicted $IC_{50}$ distributions were compared between the high- and low-TAMP groups defined from patient-level out-of-fold estimates. Lower predicted $IC_{50}$ values were interpreted as greater computationally inferred drug sensitivity. The analysis included compounds targeting cell-cycle-related mechanisms as well as agents with different therapeutic targets to determine whether the observed associations were selective rather than indicative of a generalized shift in predicted drug response.

These estimates were treated strictly as computational predictions of potential therapeutic susceptibility. They do not represent experimentally measured drug sensitivity, observed treatment response, or evidence of clinical benefit. Accordingly, the drug-response analysis was used to generate testable hypotheses regarding pharmacological vulnerabilities associated with the TAMP phenotype rather than to support treatment selection.

### Transcription-factor activity inference

Transcription-factor activity was investigated using pySCENIC and DoRothEA.^54^ pySCENIC was used to reconstruct transcriptional regulatory programs from gene-expression patterns and transcription factor-target relationships, whereas DoRothEA provided curated regulons for estimating transcription-factor activity from the expression of downstream target genes.

For transcription factor k in patient i, an activity score $A_{\mathrm{ik}}$ was inferred from the coordinated expression pattern of genes belonging to the corresponding regulon. These activity estimates were compared between the high- and low-TAMP groups defined from patient-level out-of-fold predictions. The analysis focused on identifying regulatory programs associated with differences in the image-derived mitotic phenotype, including transcription factors involved in cell-cycle progression, proliferation, differentiation, and growth control.

TF-target relationships were visualized using Cytoscape to support interpretation of the regulatory patterns associated with TAMP. Because transcription-factor activity was inferred computationally from transcriptomic profiles rather than measured directly, these estimates were interpreted as regulatory associations and not as direct evidence of transcription-factor activation.

### Statistical and survival analysis

All statistical analyses were performed in R version 4.3.0. Associations between continuous TAMP values and clinicopathological or molecular variables were evaluated using Spearman's rank correlation coefficient. Comparisons between two independent groups were performed using the Mann–Whitney *U* test, whereas variables with three or more categories were evaluated using the Kruskal–Wallis test.

Overall survival was defined as the primary clinical endpoint. For group-based survival analyses, patients were classified as high or low TAMP according to the cohort median. Survival distributions were estimated using the Kaplan–Meier method and compared with the log-rank test.

The association between TAMP and overall survival was first examined using univariable Cox proportional-hazards regression:

(8) $$\begin{array}{cccc} & h\left(t|X\right)=h_{0}\left(t\right)\exp\left(\mathrm{\beta X}\right) & & \end{array}$$

where $h\left(t|X\right)$ denotes the hazard at time t, $h_{0}\left(t\right)$ is the baseline hazard, and X represents TAMP group membership. The corresponding hazard ratio was calculated as:

(9) $$\begin{array}{cccc} & \mathrm{HR}=e^{\beta }. & & \end{array}$$

A multivariable Cox proportional-hazards model was subsequently fitted to determine whether the association between TAMP, and overall survival persisted after adjustment for available clinical prognostic factors. The model included TAMP group, age, sex, smoking status, tumor stage, and Eastern Cooperative Oncology Group (ECOG) performance status. Age was categorized as ≥65 versus <65 years, tumor stage as stages III–IV versus I–II, and ECOG performance status as ≥1 versus 0. Low TAMP, age <65 years, female sex, no smoking history, stages I–II, and ECOG 0 served as the corresponding reference categories. Adjusted hazard ratios, 95% CIs, and two-sided p-values were reported for each covariate.

The proportional-hazards assumption was assessed using Schoenfeld residuals. No statistically significant violation was detected at the 0.05 level. For analyses involving multiple simultaneous comparisons, including transcriptomic pathway enrichment, predicted drug-response analysis, and transcription-factor activity, the Benjamini–Hochberg procedure was used to control the false-discovery rate. Statistical findings were interpreted together with effect sizes and the exploratory or confirmatory nature of each analysis rather than on the basis of p-values alone.

### Computational environment

Model development, training, and inference were performed on Ubuntu 22.04 using an NVIDIA A100 GPU with 40 GB of memory and 128 GB of system RAM. The deep-learning pipeline was implemented in Python 3.9.18 with PyTorch 2.0.1 and CUDA 11.8.

Statistical and molecular analyses were conducted in R version 4.3.0. The main software packages used throughout the workflow included Albumentations 1.3.1 for image augmentation, DESeq2 1.38.3 for differential-expression analysis, clusterProfiler 4.6.0 for pathway-enrichment analysis, pySCENIC 0.12.1 and DoRothEA 1.9.0 for transcription-factor activity inference, Cytoscape 3.10.0 for regulatory-network visualization, and the survival package 3.5–5 for time-to-event analyses. The same computational environment and software versions were retained across the primary analyses to maintain consistency and reproducibility.

## Results

### ResNet-50 model performance and target-domain evaluation

The MIDOG 2022 development dataset contained 11,606 mitosis-positive patches and 351,142 mitosis-negative patches. Of the positive patches, 10,500 were assigned to the training set and 1106 to the validation set. Because of the substantial class imbalance, 50,000 negative patches were randomly sampled as non-mitotic training controls to reduce computational burden. Source-domain performance was evaluated on an independent held-out MIDOG test set of 2000 patches, comprising 821 mitotic and 1179 non-mitotic examples. For target-domain adaptation, 478 TCGA-LUSC patients contributing 512 diagnostic WSIs were retained after quality control. The cohort composition and evaluation strategy are summarized in Table 1.

**Table 1:** Cohort composition and data partitioning used for model development and evaluation.

Cohort	Patients/Cases	Slides	Positive patches	Negative patches	Train/Validation/Test
MIDOG 2022	150	150	11,606 in train/validation pool	351,142 available in train/validation pool	10,500 positive training/1106 positive validation; separate held-out test set containing 821 positive and 1179 negative patches (n=2,000)
TCGA-LUSC	478	512	Not enumerated in source data	Not enumerated in source data	Initial 70%/15%/15% patch-level split; leakage-mitigated patient-level stratified 5-fold cross-validation; additional 20% WSI-level holdout (n=102 slides)

On the held-out MIDOG test set, the ResNet-50 model achieved an AUC of 0.975 (95% CI: 0.965–0.980). At the Youden-optimal operating threshold, the classifier produced 811 true-positive, 10 false-negative, 189 false-positive, and 990 true-negative predictions.

As summarized in Table 1 and Fig. 1, the model achieved a sensitivity of 0.988, specificity of 0.840, precision of 0.811, F1-score of 0.891, and accuracy of 0.901. The error profile was characterized by very few false-negative predictions, whereas most misclassifications arose from non-mitotic patches incorrectly assigned to the mitotic class. When the MIDOG-trained ResNet-50 model was transferred directly to TCGA-LUSC without target-domain adaptation, the AUC was 0.82. Fine-tuning on TCGA-LUSC increased the initial patch-level validation AUC to 0.92. However, subsequent examination of patient identifiers revealed that 142 of 478 patients (29.7%) contributed patches to more than one data partition, including 67 patients (14.0%) represented in both the training and test sets. Because such overlap could inflate the apparent target-domain performance, we subsequently performed stratified 5-fold cross-validation at the patient level to ensure a patient-independent evaluation. Grad-CAM analysis further showed that model activation was predominantly concentrated around morphologically plausible mitotic structures, supporting the biological relevance of the learned image features.

**Fig. 1:**
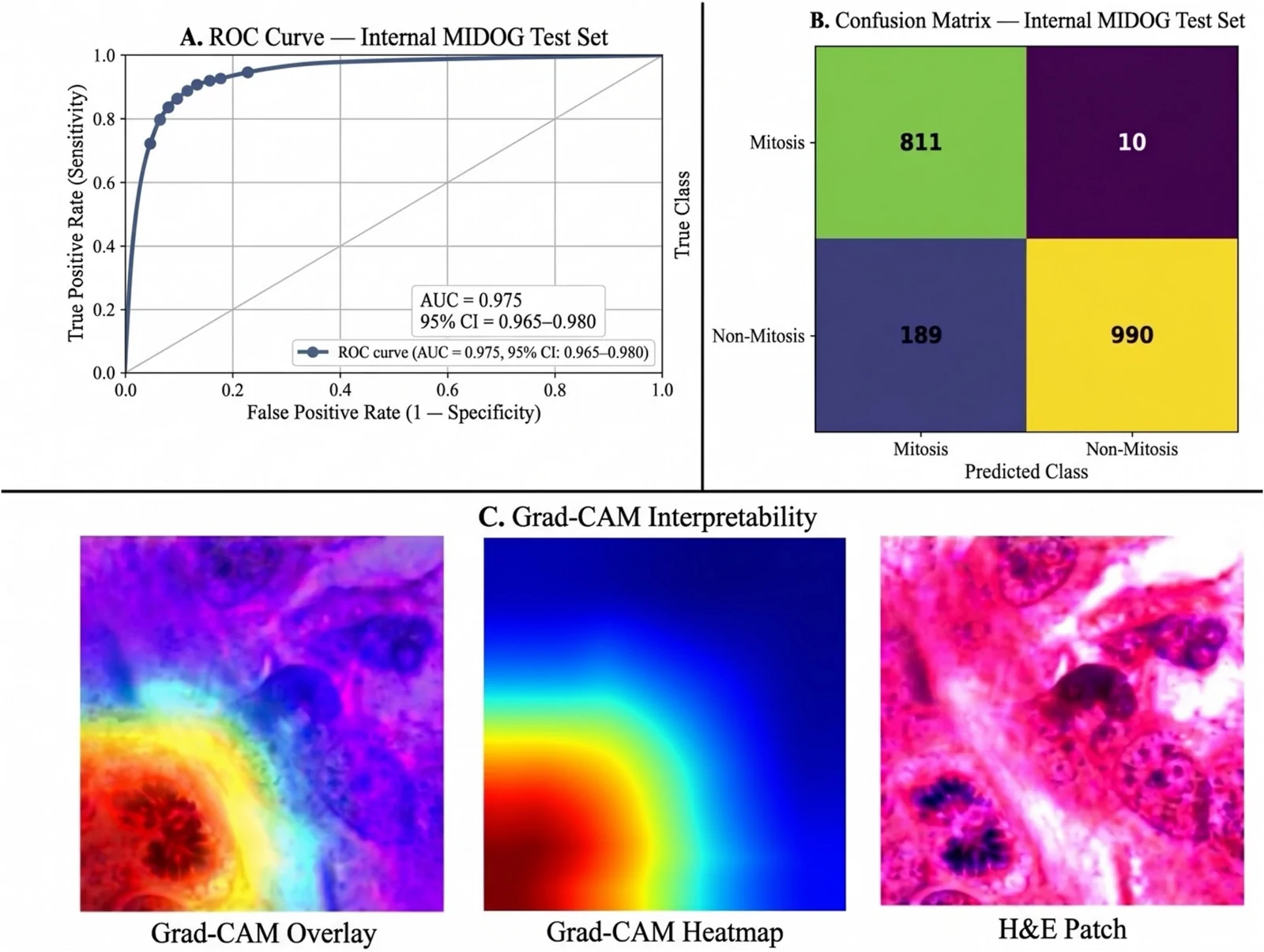
ResNet-50 model performance and interpretability on the internal MIDOG test set (n=2,000 image patches). (A) Receiver operating characteristic curve for patch-level mitosis classification, yielding an AUC of 0.975 (95% CI: 0.965–0.980). (B) Confusion matrix at the Youden-optimal operating threshold, showing 811 true positives, 10 false negatives, 189 false positives, and 990 true negatives. (C) Representative Grad-CAM heatmaps showing that model activation is concentrated on mitotic figures.

Under this leakage-mitigated design, the ResNet-50 model achieved a mean AUC of 0.89 across held-out patient folds (95% CI: 0.86–0.90). The absolute reduction of 0.05 relative to the original patch-level AUC of 0.92 indicates that patient overlap contributed modestly to the initial performance estimate. Importantly, the patient-level AUC remained higher than the pre-fine-tuning TCGA-LUSC AUC of 0.82, indicating that the improvement following target-domain adaptation was not explained solely by overlap between the original partitions.

An additional WSI-level holdout analysis was performed as a complementary robustness assessment. In this analysis, 20% of the slides (n=102) were excluded from model training. The resulting AUC was 0.90 (95% CI: 0.84–0.90), which was similar to the patient-level cross-validation estimate. Because the WSI-level analysis did not constitute the primary patient-independent validation, the patient-level 5-fold cross-validation remained the principal estimate of generalization performance.

All subsequent TAMP-based survival and molecular analyses were therefore based on patient-level out-of-fold predictions generated by the ResNet-50 model rather than predictions obtained from the original patch-level split. Grad-CAM analysis further showed that regions of high model activation were concentrated around morphologically plausible mitotic structures, providing qualitative evidence that the classifier relied on relevant nuclear morphology.

### Survival analysis

The association between TAMP and overall survival was evaluated using patient-level out-of-fold TAMP estimates generated by the ResNet-50 model within the 5-fold cross-validation framework. Patients were stratified into high- and low-TAMP groups according to the cohort median. Kaplan–Meier analysis showed significantly poorer overall survival in the high-TAMP group (log-rank p=0.008; Fig. 2). At approximately 7.5 years, the estimated overall survival probability was about 52% in the low-TAMP group and approximately 22% in the high-TAMP group. (See Fig. 3.)

**Fig. 2:**
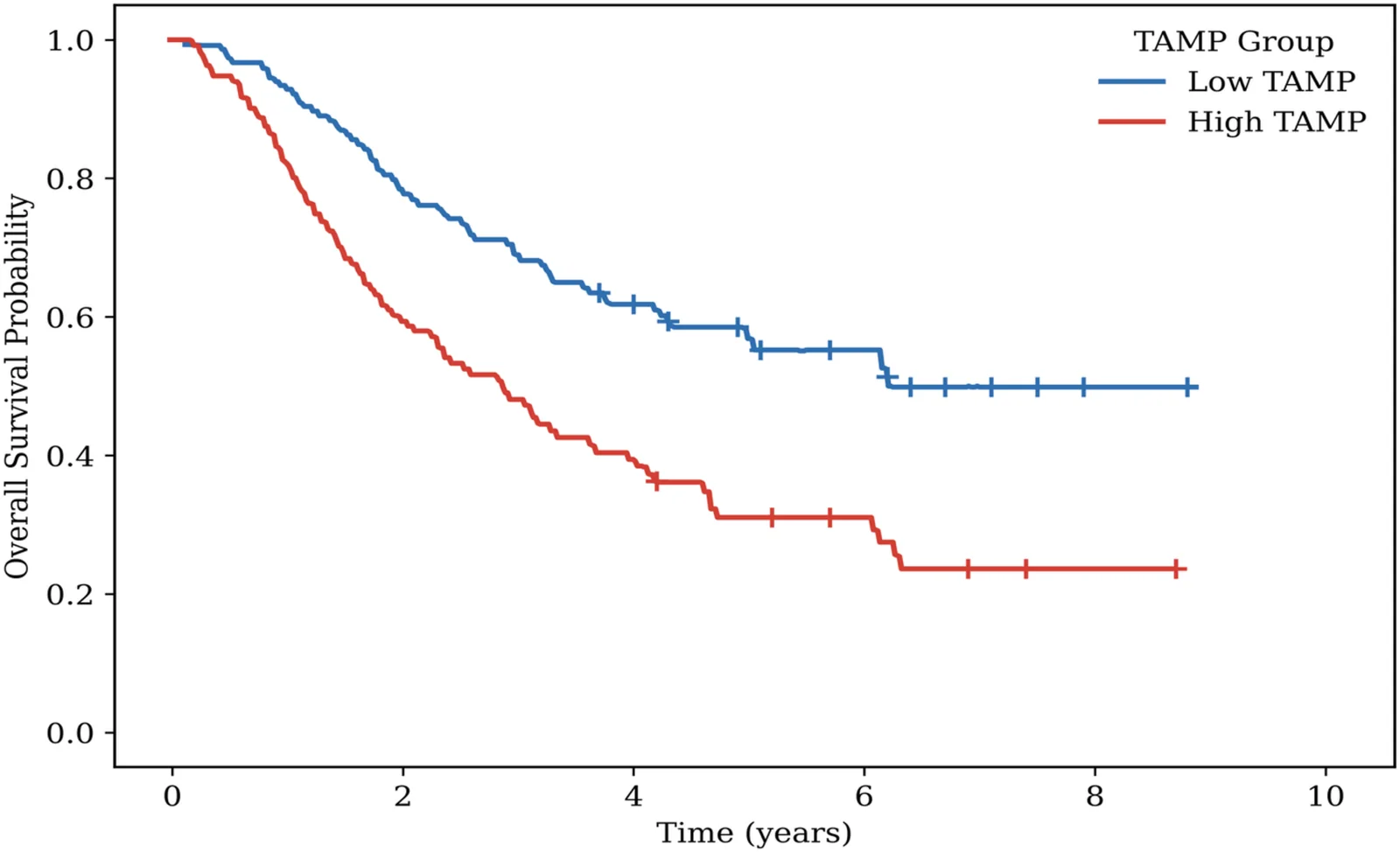
Kaplan–Meier survival analysis and Cox regression for patient-level out-of-fold TAMP estimates. Kaplan–Meier curves are stratified by high versus low TAMP groups, showing significantly poorer overall survival (OS) in the high-TAMP group (log-rank p=0.008). Univariable Cox regression yielded a hazard ratio (HR) of 1.82 (95% CI: 1.18–2.81), corresponding to an 82% higher hazard of death in the high-TAMP group. In multivariable analysis adjusted for age, sex, smoking status, tumor stage, and ECOG performance status, high TAMP remained significantly associated with poorer OS (adjusted HR = 1.68, 95% CI: 1.08–2.62; p=0.022).

**Fig. 3:**
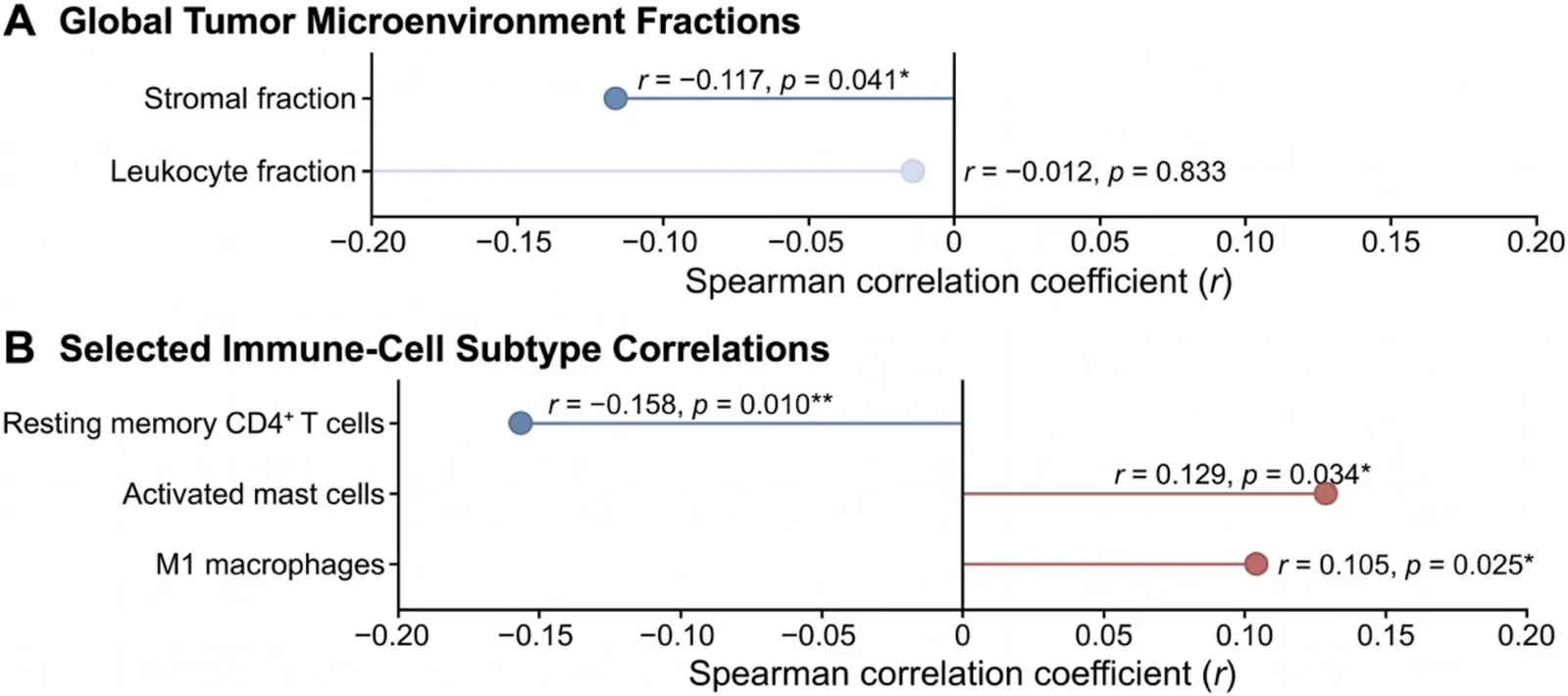
Associations between tumor-area mitosis proportion (TAMP) and selected components of the tumor microenvironment in the TCGA-LUSC cohort. TAMP was negatively correlated with stromal fraction $\left(r^{\prime }=-0.117,p=0.041\right)$ and resting memory $\mathrm{CD}4^{+}$T cells $\left(r^{\prime }=-0.158,p=0.010\right)$, whereas activated mast cells $\left(r^{\prime }=0.129,p=0.034\right)$ and M1 macrophages $\left(r^{\prime }=0.105,p=0.025\right)$ showed modest positive correlations. Overall leukocyte fraction, CD8^+^ T cells, and regulatory T cells were not significantly associated with TAMP.

Univariable Cox proportional-hazards regression showed that high TAMP was associated with increased mortality risk. Compared with the low-TAMP group, patients with high TAMP had a HR of 1.82 (95% CI: 1.18–2.81, p<0.01), corresponding to an 82% higher hazard of death during follow-up. The proportional-hazards assumption was assessed using Schoenfeld residuals, with no statistically significant violation detected at the 0.05 level.

A multivariable Cox model was subsequently fitted to determine whether this association persisted after adjustment for clinical prognostic factors. High TAMP remained associated with poorer overall survival after adjustment for age, sex, smoking status, tumor stage, and ECOG performance status (adjusted HR = 1.68, 95% CI: 1.08–2.62, p=0.022). Advanced tumor stage was also associated with poorer survival (HR = 1.89, 95% CI: 1.34–2.67, p<0.001), whereas ECOG performance status ≥1 showed a weaker association (HR = 1.42, 95% CI: 1.01–2.00, p=0.045). Age, sex, and smoking status were not statistically significant in the adjusted model.

Because TAMP was derived from out-of-fold predictions, each patient's survival analysis used a TAMP estimate generated by a model that had not been trained on image patches from that patient. The persistence of the survival association after multivariable adjustment indicates that TAMP retained prognostic information beyond the clinical variables included in the model, although external validation is required before broader independent prognostic value can be established.

### Tumor microenvironment analysis

The association between patient-level out-of-fold TAMP estimates and the tumor microenvironment was examined across stromal and immune compartments. TAMP showed a modest negative correlation with stromal fraction $\left(r^{\prime }=-0.117,p=0.041\right)$, whereas no association was observed with overall leukocyte fraction $\left(r^{\prime }=-0.012,p=0.833\right)$.

At the immune-cell subtype level, resting memory $\mathrm{CD}4^{+}$T-cell fraction was negatively correlated with TAMP $\left(r^{\prime }=-0.158,p=0.010\right)$. In contrast, activated mast cells $\left(r^{\prime }=0.129,p=0.034\right)$ and M1 macrophages $\left(r^{\prime }=0.105,p=0.025\right)$ showed modest positive correlations. No statistically significant associations were observed for CD8^+^ T cells (r=−0.089, p=0.112) or regulatory T cells (r=0.076, p=0.187). Taken together, these findings indicate that variation in TAMP is associated with selective differences in stromal and immune-cell composition rather than with a generalized change in leukocyte infiltration.

### Mutation analysis

The association between patient-level out-of-fold TAMP estimates and genomic characteristics was examined to determine whether the image-derived mitotic phenotype was related to specific patterns of genomic alteration. TAMP showed a positive correlation with indel-derived neoantigen load (r=0.158,p=0.007; Fig. 4) and with overall indel burden $\left(r^{\prime }=0.121,p=0.038\right)$.

**Fig. 4:**
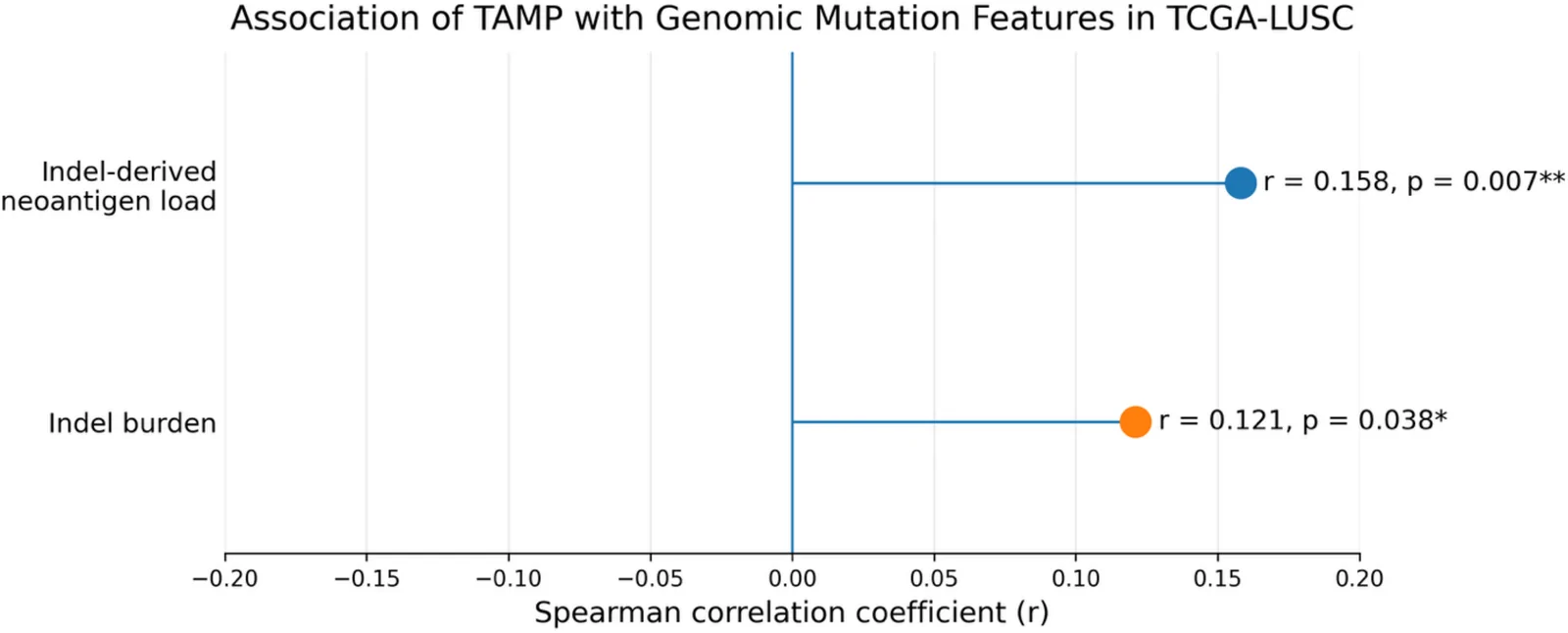
Associations between tumor-area mitosis proportion (TAMP) and selected genomic characteristics in the TCGA-LUSC cohort. TAMP showed positive correlations with indel-derived neoantigen load $\left(r^{\prime }=0.158,p=0.007\right)$ and overall indel burden $\left(r^{\prime }=0.121,p=0.038\right)$. Associations with SNV-derived neoantigen load, mutation-rate measures, chromosomal segmentation, and chromosomal fragmentation were not statistically significant.

By contrast, no statistically significant associations were observed with SNV-derived neoantigen load (r=0.094, p=0.104), silent mutation rate (r=0.087, p=0.132), non-silent mutation rate (r=0.102, p=0.079), chromosomal segmentation (r=0.078, p=0.178), or chromosomal fragmentation (r=0.065, p=0.256). The pattern therefore suggests that the relationship between TAMP and genomic variation is selective rather than global. The significant associations were confined primarily to indel-related measures, whereas broader indicators of mutation rate and chromosomal alteration showed weak, non-significant relationships.

These findings indicate that TAMP should not be interpreted as a surrogate for generalized genomic instability. Rather, the image-derived phenotype appears to capture a limited component of genomic variation associated with indel-related tumor characteristics.

### Pathway enrichment analysis and molecular associations

Gene set enrichment analysis was used to characterize coordinated transcriptional programs associated with patient-level out-of-fold TAMP. Patients were divided into high- and low-TAMP groups using the cohort median, and genes were ranked according to differential-expression statistics. Positive enrichment scores therefore indicate preferential representation toward the high-TAMP end of the ranked gene list.

The REGULATION_STEM_CELL_PROLIFERATION gene set was enriched in high-TAMP tumors (ES = 0.555, NES = 2.14, FDR < 0.001; Fig. 5A). Leading-edge genes included MYC and components associated with WNT/β-catenin signaling, linking elevated TAMP to transcriptional programs involved in proliferation and stem-cell regulation. In contrast, differentiation-associated genes, including members of the BMP family, were preferentially represented toward the low-TAMP end of the ranked profile.

**Fig. 5:**
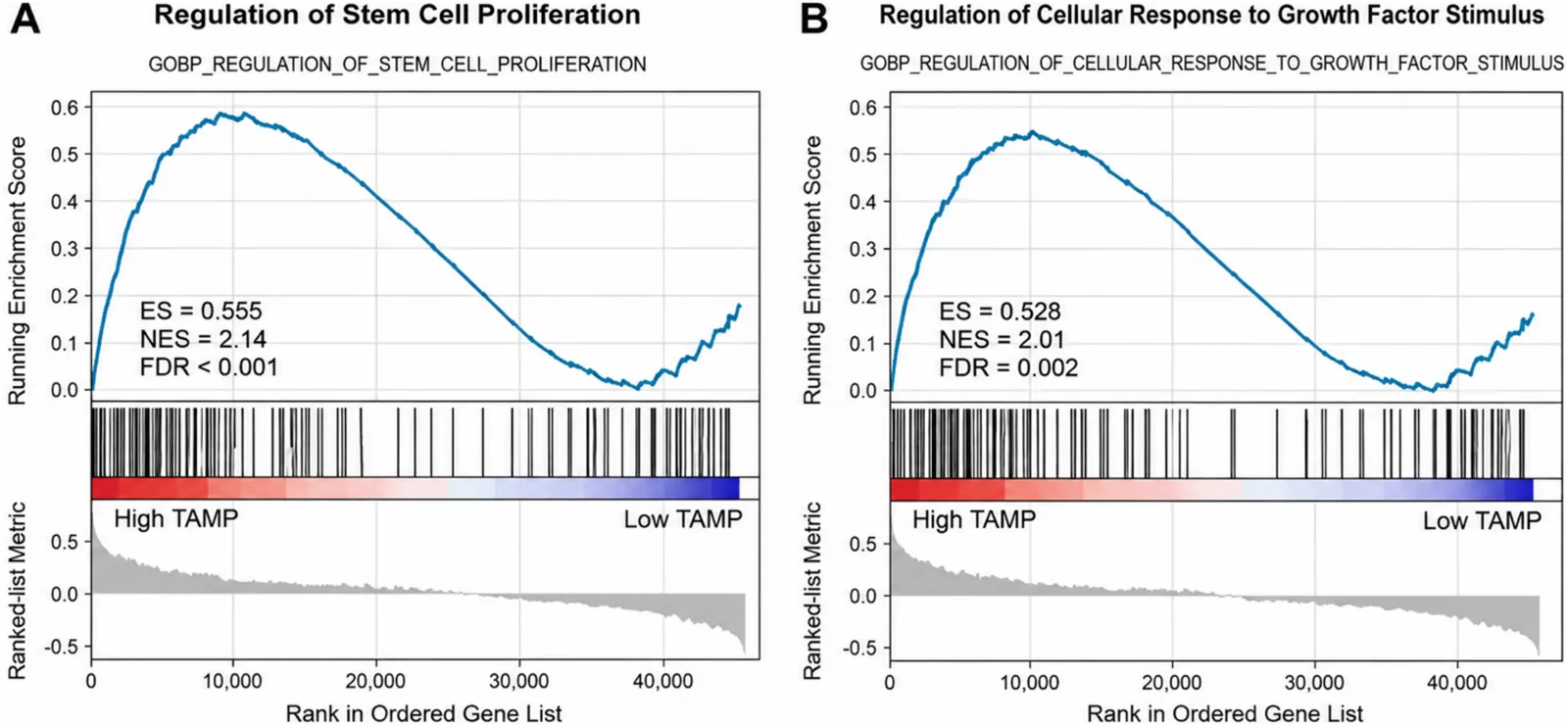
Pathway enrichment associated with TAMP in the TCGA-LUSC cohort. (A) REGULATION_STEM_CELL_PROLIFERATION was enriched in high-TAMP tumors (ES = 0.555, NES = 2.14, FDR < 0.001). (B) REGULATION_GROWTH_FACTOR_RESPONSE was enriched in the high-TAMP group (ES = 0.528, NES = 2.01, FDR = 0.002). Both gene sets showed positive enrichment in tumors with elevated TAMP.

REGULATION_GROWTH_FACTOR_RESPONSE was also enriched in high-TAMP tumors (ES = 0.528, NES = 2.01, FDR = 0.002; Fig. 5B). Genes contributing to this enrichment included components related to MAPK and PI3K-AKT signaling, indicating that the high-TAMP phenotype was accompanied by broader transcriptional responses associated with growth-factor signaling.

Additional enrichment was observed for E2F_TARGETS (ES = 0.512, NES = 1.96, FDR = 0.003), G2M_CHECKPOINT (ES = 0.498, NES = 1.91, FDR = 0.005), and MYC_TARGETS_V1 (ES = 0.487, NES = 1.87, FDR = 0.008). The enrichment of E2F- and G2M-related programs provides further evidence that elevated TAMP is associated with transcriptional activity involving cell-cycle progression and mitotic checkpoint regulation. MYC-target enrichment is consistent with the broader proliferative profile observed in tumors with high TAMP.

Taken together, these results indicate that high TAMP is associated with a coordinated transcriptional state involving stem-cell regulation, growth-factor response, E2F signaling, G2M checkpoint activity, and MYC-associated programs. These findings provide molecular context for the image-derived phenotype but should be interpreted as pathway-level associations. GSEA does not establish functional pathway activation or a causal relationship between mitotic morphology and the corresponding signaling programs.

### Predicted drug response and inferred transcription-factor activity associated with TAMP status

Predicted drug-response profiles were compared between high- and low-TAMP tumors using patient-level out-of-fold TAMP estimates. High-TAMP tumors showed lower predicted $IC_{50}$values for palbociclib, alisertib, and AZD7762 (all p<0.001, FDR < 0.001; Fig. 6A). Dinaciclib showed a similar pattern (p=0.002, FDR = 0.004). In contrast, predicted responses to erlotinib (p=0.521, FDR = 0.521) and cisplatin (p=0.387, FDR = 0.516) did not differ significantly between the TAMP groups. The drug-response pattern was therefore selective rather than uniform across the evaluated compounds and was most evident for agents targeting cell-cycle-related mechanisms.

**Fig. 6:**
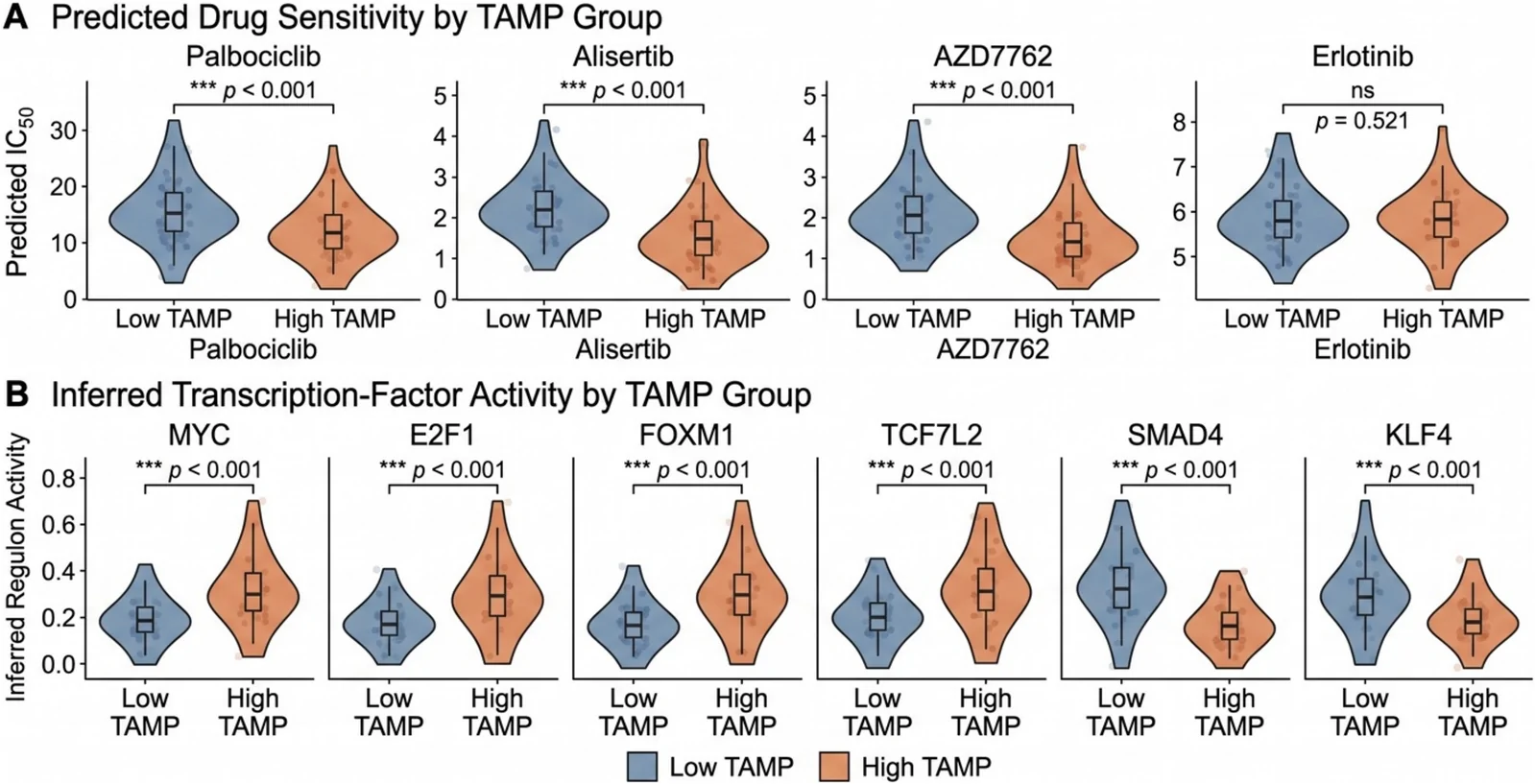
Predicted drug response and inferred transcription-factor activity according to TAMP status in TCGA-LUSC. (A) High-TAMP tumors showed lower predicted $IC_{50}$ values for palbociclib, alisertib, and AZD7762 (all p<0.001), whereas erlotinib showed no significant difference (p=0.521). (B) MYC, E2F1, FOXM1, and TCF7L2 showed higher inferred activity in high-TAMP tumors, whereas SMAD4 and KLF4 showed lower inferred activity (all p<0.001).

Inferred transcription-factor activity also differed between the two TAMP groups (Fig. 6B). MYC, E2F1, FOXM1, and TCF7L2 showed higher inferred activity in high-TAMP tumors (all p<0.001, FDR < 0.001), whereas SMAD4 and KLF4 showed lower activity (all p<0.001, FDR < 0.001). TP53 activity was also lower in the high-TAMP group (p=0.003, FDR = 0.006). The increased inferred activity of MYC, E2F1, and FOXM1 was consistent with the cell-cycle and proliferation-related pathway enrichment described in the Pathway enrichment analysis and molecular associations section, whereas lower SMAD4, KLF4, and TP53 activity indicated additional differences in regulatory programs related to differentiation and growth control.

Taken together, these analyses indicate that elevated TAMP was accompanied by a selective pattern of predicted sensitivity to cell-cycle-targeting compounds and by distinct transcriptional regulatory activity related to proliferation. These findings should nevertheless be interpreted cautiously because both drug response and transcription-factor activity were inferred computationally from transcriptomic data. Predicted $IC_{50}$ values do not represent experimentally measured treatment responses, and inferred transcription-factor scores do not constitute direct biochemical measurements of regulatory activity. The observed associations are therefore hypothesis-generating and require experimental validation before therapeutic implications can be established.

The selectivity of these findings provides additional biological context. High TAMP was not associated with uniformly lower predicted $IC_{50}$ values across all evaluated compounds, nor with a generalized increase in transcription-factor activity. Instead, the strongest differences were concentrated in cell-cycle-related agents and regulatory programs linked to proliferation. This pattern was consistent with the enrichment of E2F-target, G2M-checkpoint, and MYC-associated transcriptional programs and supports the interpretation of TAMP as a marker of a coordinated proliferative tumor state rather than a non-specific molecular disturbance. The absence of corresponding differences for erlotinib and cisplatin further indicates that the predicted pharmacological signal was not broadly distributed across unrelated therapeutic mechanisms.

## Discussion

The present study shows that mitosis-related image features learned in a source tumor domain can be adapted to LUSC while retaining meaningful discrimination under patient-independent evaluation. The classifier achieved an AUC of 0.975 on the held-out MIDOG test set, and target-domain fine-tuning increased the initial TCGA-LUSC patch-level AUC from 0.82 to 0.92. Because the original patch-level partition allowed images from the same patient to appear in different subsets, performance was re-assessed using patient-level 5-fold cross-validation. Under this stricter design, the mean AUC was 0.89 (95% CI: 0.86–0.90), whereas the complementary WSI-level holdout analysis yielded an AUC of 0.90 (95% CI: 0.84–0.90). The decrease from the initial patch-level estimate confirms that patient overlap introduced some optimistic bias, but the patient-independent performance remained clearly above the pre-fine-tuning AUC of 0.82. The observed improvement therefore cannot be attributed solely to overlap between the original partitions and is consistent with the known sensitivity of computational pathology models to tissue morphology, staining variation, and image-acquisition conditions.^16^, ^27^, ^28^

The error profile is also relevant to the interpretation of TAMP. On the MIDOG test set, the classifier missed only 10 mitotic patches but incorrectly classified 189 non-mitotic patches as positive, indicating an operating point that favored sensitivity. Such behavior is reasonable for identifying sparse mitotic events, but it can increase the number of positive predictions when the classifier is applied over large tumor regions. TAMP should therefore be interpreted as the proportion of evaluable tumor patches exhibiting model-detected mitotic morphology rather than as a direct substitute for a manually enumerated mitotic count per high-power field. This distinction is important because TAMP summarizes the spatial prevalence of model-positive regions rather than the absolute number of individual mitotic figures. All downstream analyses were based on patient-level out-of-fold predictions, so each patient's TAMP was generated by a model that had not been trained on patches from that patient. Grad-CAM localization around morphologically plausible mitotic structures provided additional qualitative support that the classifier relied on relevant nuclear features.

The survival analysis provided additional clinical context for the image-derived phenotype. Patients with high TAMP had significantly poorer overall survival than those with low TAMP (log-rank p=0.008). In univariable Cox analysis, high TAMP was associated with an HR of 1.82 (95% CI: 1.18–2.81). Importantly, the association persisted after adjustment for age, sex, smoking status, tumor stage, and ECOG performance status (adjusted HR = 1.68, 95% CI: 1.08–2.62; p=0.022). Advanced tumor stage was also associated with poorer survival, whereas ECOG performance status showed a weaker but statistically significant association. The persistence of the TAMP effect after adjustment indicates that the observed survival relationship was not explained entirely by the clinical factors included in the model. The direction of this association is biologically consistent with the established relationship between mitotic activity and aggressive tumor behavior and with the use of mitotic assessment in grading or classification systems for other malignancies.^9^, ^10^ Nevertheless, the analysis remains retrospective, and the TAMP threshold was derived from the same cohort in which its prognostic association was evaluated. The adjusted result should therefore be regarded as evidence of prognostic information within TCGA-LUSC rather than as proof of independently validated clinical utility.

The tumor-microenvironment analysis suggested selective rather than generalized relationships between TAMP and immune or stromal composition. TAMP was negatively correlated with stromal fraction (r=−0.134, p=0.012) but showed no association with overall leukocyte fraction (r=−0.015, p=0.781). Resting memory CD4+ T cells were negatively associated with TAMP (r=−0.172, p=0.005), whereas activated mast cells (r=0.142, p=0.018) and M1 macrophages (r=0.118, p=0.031) showed modest positive relationships. By contrast, CD8+ T cells and regulatory T cells were not significantly associated with TAMP. The small effect sizes argue against interpreting high TAMP as a broadly immune-enriched phenotype. Rather, the findings indicate limited shifts in selected stromal and immune-cell populations. Previous computational pathology studies have shown that histological organization can encode aspects of immune and molecular tumor state^39^, ^44^; the present results suggest that a mitosis-related imaging phenotype captures only a modest component of this variation.

The genomic findings showed a similarly selective pattern. TAMP was positively correlated with indel-derived neoantigen load (r=0.158, p=0.007) and overall indel burden (r=0.121, p=0.038). Associations with SNV-derived neoantigen load, silent and non-silent mutation rates, chromosomal segmentation, and chromosomal fragmentation were not statistically significant. These findings do not support TAMP as a surrogate for generalized genomic instability. Instead, they suggest that mitosis-associated morphology carries limited information related to selected indel-associated characteristics. This interpretation is consistent with previous evidence that histological patterns in NSCLC can contain probabilistic information about underlying molecular alterations.^39^

The transcriptomic results provided a more coherent biological context for the high-TAMP phenotype. In addition to significant enrichment of REGULATION_STEM_CELL_PROLIFERATION (ES = 0.555, FDR < 0.001) and REGULATION_GROWTH_FACTOR_RESPONSE (ES = 0.528, FDR = 0.002), high-TAMP tumors showed enrichment of E2F_TARGETS (ES = 0.512, FDR = 0.003), G2M_CHECKPOINT (ES = 0.498, FDR = 0.005), and MYC_TARGETS_V1 (ES = 0.487, FDR = 0.008). These signatures converge on biological processes closely related to cell-cycle progression, mitotic control, proliferative capacity, and growth signaling. The preferential representation of differentiation-associated genes, including BMP-family members, toward the low-TAMP end of the ranked profile further indicates that the two TAMP groups differed in broader transcriptional state. The enrichment findings therefore support the interpretation of TAMP as an image-derived phenotype associated with a proliferative molecular program. They do not, however, demonstrate that increased mitotic morphology directly activates the corresponding pathways.

The inferred transcription-factor profiles were consistent with this interpretation. MYC, E2F1, FOXM1, and TCF7L2 showed higher inferred activity in high-TAMP tumors, whereas SMAD4, KLF4, and TP53 showed lower activity. The agreement between the E2F-, MYC-, and cell-cycle-related pathway results and the corresponding transcription-factor estimates provides internal biological consistency across the transcriptomic analyses. In particular, increased inferred activity of E2F1 and FOXM1 accords with the enrichment of E2F-target and G2M-checkpoint programs. These estimates remain computational inferences derived from transcriptomic data and should not be interpreted as direct biochemical measurements of regulatory activity.

Predicted drug-response analysis provided an additional, but explicitly hypothesis-generating, layer of evidence. High-TAMP tumors showed lower predicted $IC_{50}$values for palbociclib, alisertib, AZD7762, and dinaciclib, whereas erlotinib and cisplatin showed no significant differences between TAMP groups. The selective pattern is notable because the compounds associated with lower predicted $IC_{50}$ values primarily target cell-cycle-related mechanisms, which is consistent with the pathway and transcription-factor findings. However, these estimates were generated computationally using oncoPredict^46^ and do not constitute experimentally observed drug sensitivity or patient treatment response. They should therefore be regarded as candidate pharmacological relationships for future experimental investigation rather than evidence supporting therapeutic selection.

Taken together, the analyses indicate that TAMP captures a mitosis-related imaging phenotype with different levels of association across clinical and molecular data layers. The survival and transcriptomic findings were the most consistent, whereas immune and genomic associations were weaker and more selective. This distinction is important because statistical significance across several data types does not imply equivalent biological importance or clinical relevance. The results are more appropriately interpreted as evidence that the spatial prevalence of model-detected mitotic morphology reflects a broader proliferative state that can be observed at histological, transcriptomic, regulatory, and clinical levels.

The principal contribution of this work therefore extends beyond patch-level mitosis classification. Previous studies have established the technical feasibility of deep-learning-based mitosis detection^13^, ^14^, ^16^, ^34^, ^35^, ^36^, ^37^, ^38^ and have shown that WSI representations can encode clinically and molecularly relevant information.^39^, ^40^, ^41^, ^43^ Here, these two directions are connected through a directly interpretable histomorphological phenotype. By deriving TAMP from patient-level out-of-fold predictions and examining it alongside survival, immune, genomic, transcriptomic, regulatory, and predicted pharmacological characteristics, the framework places automated mitosis analysis within a broader patient-level biological context.

Several limitations should be considered when interpreting these findings. Model evaluation remained internal to TCGA-LUSC, the patch-based design provided limited representation of broader tissue architecture, and the individual contributions of the transfer-learning and augmentation components were not isolated through formal ablation analyses. In addition, several downstream molecular analyses relied on observational data or computational inference. These issues are discussed in greater detail in the Limitations section. Overall, the results indicate that TAMP is associated with poorer overall survival and with a proliferative molecular state in LUSC. Patient-level cross-validation showed that substantial target-domain discrimination was retained after correcting for overlap in the initial patch-level partition, and the survival association persisted after adjustment for available clinical prognostic factors. The findings support further investigation of TAMP as a research-derived imaging phenotype, but external patient-level validation, prespecified threshold assessment, evaluation of incremental prognostic value, and experimental confirmation of the molecular and pharmacological associations remain necessary before clinical application.

## Limitations

This study has several limitations. First, the retrospective design and reliance on the TCGA-LUSC cohort may limit generalizability. Although potential leakage from the initial patch-level partition was mitigated using patient-level 5-fold cross-validation and further assessed through a WSI-level holdout analysis, all evaluations were derived from the same overall source cohort. External validation is therefore required to determine whether the observed performance is preserved across independent patient populations, tissue-processing workflows, staining conditions, and scanning platforms.

Second, the multivariable Cox model included age, sex, smoking status, tumor stage, and ECOG performance status. However, several potentially relevant clinical factors, including treatment exposure, comorbidities, detailed smoking history, and molecularly guided therapies, were not comprehensively available. Moreover, the median TAMP threshold used for survival stratification was derived from the same cohort in which prognostic associations were evaluated. Validation using an independently predefined threshold and external clinical cohorts would provide a more rigorous assessment of the incremental prognostic value of TAMP beyond established clinicopathological variables.

Third, the use of 128×128-pixel patches emphasizes local nuclear morphology but provides limited representation of broader tissue architecture, spatial organization, and intratumoral heterogeneity. Multiscale or spatially informed modeling strategies may therefore complement the current approach. In addition, the individual contributions of Focal Loss, HSV augmentation, source-domain transfer learning, and selective layer freezing were not quantified through systematic ablation experiments. Such analyses would help clarify which components contributed most to the observed performance gains.

Finally, the genomic, transcriptomic, immune, pathway, transcription-factor, and drug-response analyses were based on observational data or computational inference. Accordingly, these results should be interpreted as associative evidence that provides biological context for the TAMP phenotype rather than as evidence of direct causal relationships. In particular, inferred transcription-factor activity and predicted drug sensitivity represent computational estimates rather than direct biochemical or clinical measurements. Independent cohort validation, together with targeted experimental assessment of selected molecular and pharmacological findings, will be necessary to strengthen the biological interpretation and translational relevance of TAMP.

## Conclusions

This study developed and internally evaluated a transfer-learning framework for characterizing mitosis-positive tumor regions in LUSC. The classifier achieved an AUC of 0.975 on the held-out MIDOG test set. After transfer to TCGA-LUSC, fine-tuning increased the initial patch-level AUC from 0.82 to 0.92. When patient overlap was removed through stratified 5-fold patient-level cross-validation, the mean AUC was 0.89 (95% CI: 0.86–0.90). A complementary WSI-level hold-out analysis yielded an AUC of 0.90 (95% CI: 0.84–0.90). The lower estimates obtained under patient-independent evaluation indicate that the original patch-level split was modestly optimistic, whereas the retained performance supports effective adaptation of mitosis-related image features from the source domain to LUSC.

Patient-level out-of-fold predictions were used to derive TAMP as the proportion of evaluable tumor-region patches classified as mitosis-positive. High TAMP was associated with poorer overall survival in univariable analysis (HR = 1.82, 95% CI: 1.18–2.81) and remained associated with mortality after adjustment for age, sex, smoking status, tumor stage, and ECOG performance status (adjusted HR = 1.68, 95% CI: 1.08–2.62; *p* = 0.022). Higher TAMP was also associated with selected stromal and immune-cell differences and with greater indel-derived neoantigen load and indel burden. Transcriptomic analysis showed enrichment of stem-cell proliferation, growth-factor-response, E2F-target, G2M-checkpoint, and MYC-target programs in high-TAMP tumors. Consistent differences were also observed in inferred transcription-factor activity and in predicted responses to selected cell-cycle-targeting compounds.

TAMP should be interpreted as an image-derived measure of the prevalence of mitosis-positive tumor regions rather than as a direct substitute for conventional mitotic counting. Within TCGA-LUSC, it captured clinically relevant survival information while also reflecting a broader proliferative molecular state. Its prognostic and biological interpretation nevertheless remains limited by the retrospective single-cohort design and the computational nature of several downstream analyses. Independent multicenter validation, evaluation of a prespecified TAMP threshold, formal assessment of incremental prognostic value, and experimental confirmation of the molecular and pharmacological associations are required before clinical application.

## Consent for publication

Not applicable.

## Declaration of competing interest

The authors declare that they have no known competing financial interests or personal relationships that could have appeared to influence the work reported in this article.

## Data Availability

The TCGA-LUSC diagnostic WSIs and matched clinical, genomic, and transcriptomic data analyzed in this study are publicly available through the National Cancer Institute Genomic Data Commons. The MIDOG 2022 dataset used for source-domain model development is available through the MIDOG 2022 challenge platform under the applicable data-use conditions. Derived study materials, including TCGA-LUSC annotation masks, patch coordinates, patient-level out-of-fold TAMP estimates, trained model weights, and the inference pipeline, are available from the corresponding author upon reasonable request for non-commercial research purposes, subject to applicable institutional and source-data use policies.
